# Combining computational models, semantic annotations and simulation experiments in a graph database

**DOI:** 10.1093/database/bau130

**Published:** 2015-03-08

**Authors:** Ron Henkel, Olaf Wolkenhauer, Dagmar Waltemath

**Affiliations:** ^1^University of Rostock, Department of Computer Science, Albert-Einstein-Straße 22, D-18059 Rostock, Germany, ^2^Department of Systems Biology and Bioinformatics, University of Rostock, Ulmenstrasse 69, 18057 Rostock, Germany and ^3^Stellenbosch Institute for Advanced Study (STIAS), Wallenberg Research Centre at Stellenbosch University, Stellenbosch 7600, South Africa

## Abstract

Model repositories such as the BioModels Database, the CellML Model Repository or JWS Online are frequently accessed to retrieve computational models of biological systems. However, their storage concepts support only restricted types of queries and not all data inside the repositories can be retrieved. In this article we present a storage concept that meets this challenge. It grounds on a graph database, reflects the models’ structure, incorporates semantic annotations and simulation descriptions and ultimately connects different types of model-related data. The connections between heterogeneous model-related data and bio-ontologies enable efficient search via biological facts and grant access to new model features. The introduced concept notably improves the access of computational models and associated simulations in a model repository. This has positive effects on tasks such as model search, retrieval, ranking, matching and filtering. Furthermore, our work for the first time enables CellML- and Systems Biology Markup Language-encoded models to be effectively maintained in one database. We show how these models can be linked via annotations and queried.

**Database URL:** https://sems.uni-rostock.de/projects/masymos/

## Introduction

Model repositories such as the BioModels Database (1), the CellML model repository (2) or JWS Online (3) offer to the community valuable, curated and reusable models describing biological systems. They enable researchers to study biological systems in the computer without necessarily implementing the models from scratch, thereby saving time, effort and money. In addition, curation has a positive effect on the quality of models used in modeling projects. Errors in the models’ encoding are more likely to be detected, they can be resolved and documented. Finally, models are distributed in standard formats, e.g. the Systems Biology Markup Language (SBML) (4) or CellML (5), making them immediately available to a large number of computational tools for simulation, analysis, visualization or comparison (6).

Each model describes certain aspects of a system. These aspects may be of functional, behavioral or structural nature (7) and need to be covered in the description of the model. Semantic annotations relate model entities to external resources describing the underlying biology. For example, a model of the cell cycle may be annotated with a term from Gene Ontology (GO) (8) defining the cell cycle biologically, e.g.

The progression of biochemical and morphological phases and events that occur in a cell during successive cell replication or nuclear replication events. Canonically, the cell cycle comprises the replication and segregation of genetic material followed by the division of the cell, but in endocycles or syncytial cells nuclear replication or nuclear division may not be followed by cell division. (Gene Ontology, GO:0007049)

Please note that the SBML file would only be equipped with the GO identifier (here: GO:0007049). This identifier can then be resolved computationally to access the full information from GO, making a semantic-based comparison of models feasible, e.g. (9–12).

Over the past years, the focus shifted from encoding pure model code toward providing full model-related information (13, 14). As a consequence, researchers have access to detailed descriptions of what a model is about, the rationale behind building it and ultimately how to reuse it. The necessary information to reuse a model is defined in the Minimum Information guideline for the annotation of models, MIRIAM (13). A MIRIAM-compliant model contains information about each biological entity, links to the publication describing a model (denoted as reference publication) and instructions on how to use a model to reproduce a published result. Driven by the definition of the MIRIAM requirements and related efforts under the Computational Network for Modeling in Biology [COMBINE (15)], model repositories provide richly annotated models.

Before the era of semantic knowledge integration and ontologies, model code contained only few meta-data. Models could easily be kept in file systems and meta-data in relational data tables (1). A main feature of relational data tables is their fixed database schema which enables fast storage of and search for homogeneous data items. This storage concept has become unsuitable, because today’s models contain heterogeneous meta-data. Even MIRIAM-compliant annotations cannot be mapped efficiently onto relational tables. The heterogeneous structure and content of the various meta-data encoded in a model do not comply with the homogenous and pre-defined properties of relational tables. This (technical) limitation led to a situation where many types of model-related data are not extracted from the model. Consequently, the information contained in these meta-data items is not accessible for any kind of comparison or reasoning. Meta-data that are in principle available, but not retrievable include the structure of the model (16), model versions (17) and simulation setups (18). One consequence of inaccessible meta-data is that modeling results may become irreproducible, because the information on the simulation experiment is not associated with the model code (19).

**Table 1.** Top: Number of files and stored nodes for each data domain and Bottom: Number of nodes for each stored ontology

Data domain	Documents	Nodes
SBML	462	91 488
CellML	841	143 521
SED-ML	38	3352

Ontology	Nodes	Domain references

KiSAO	261	38
SBO	606	8839
GO	39787	7555

Notes: The domain references state the number of links from a concept of an Ontology into a data domain. Here, all KiSAO concepts are linked to the domain of SED-ML whereas all SBO and GO concepts are referred to from the CellML or SBML domain.

In this manuscript, we propose the concept of graph databases for model storage and retrieval. Graph databases support heterogeneous data structures. They furthermore enable a flexible integration of model-related meta-data. We focus our studies on models in SBML and CellML formats, associated simulation setups in Simulation Experiment Description Markup Language (SED-ML) format and semantic annotations from bio-ontologies. A key feature of our work is the *explicit linking of data*. It enables, for the first time, queries across different data formats, e.g. ‘Return experiments observing entities representing an “m-phase inducer phosphatase” and acting as modifier in a reaction’. This query incorporates and links several types of meta-data and creates a complete picture of the model: model code (identifying all models that contain entity X); semantic annotations (identifying all entities X that are annotated with concepts from a bio-ontology that relate to ‘m-phase inducer phosphatase’); the model’s network structure (filtering those models where X is a modifier in a reaction); and simulation experiments (identifying possible simulation setups for the chosen models). Our concept, when implemented in open and private model repositories, supports modelers and biologists in retrieving models and scientific findings, fosters the exploration of published models and increases model reuse.

## A graph database for simulation models and associated data

Model reuse can be improved if models and meta-data are considered together. In this article, we present a novel storage concept that tightly links model code with model-related data. Our concept is directly relevant for developers of model repositories in computational biology, as it offers new possibilities for model search and comparison. Our work also points out the possibilities of state-of-the-art database techniques for handling the increasing amount of data related to a modeling work.

Our work was driven by the question: If models encode networks—why do we not store them as graphs rather than as relational tables? In a graph database, nodes contain the data and edges represent the links between the data. Graph databases are a suitable technology for the integration and storage of modeling results, because: (1) Many models in public databases encode networks that can be represented as graphs. (2) No unified schema exists for models and meta-data, making it difficult to define a relational database schema. (3) The highly linked models, model entities and meta-data are difficult to represent in a table-based relational database management system such as MySQL. Architectural choices in current model repositories date back to times when only a limited number of alternatives existed, standardization of external knowledge only began and model files were only scarcely associated with meta-data. Since then the databases have grown and functionality has been extended. The focus has shifted from model code to ‘model-related data’. Interestingly, only a few systems’ architectures have been revised.

Traditionally, relational databases were developed for homogeneous, structured data, e.g. numerical data. Models, however, take various size and structure. SBML models in BioModels Database, for example, import data structures from external standards and link to entries in bio-ontologies. Among the external standards are ‘vCard’, electronic business cards that identify the model author and curators (http://www.w3.org/TR/vcard-rdf/), or ‘Dublin Core’, a vocabulary mainly used to describe web resources (http://dublincore.org/). Some models are provided with simulation setups and graphical representations. Designing a relational representation for these links and keeping the database efficient at the same time are impossible. A core concept of relational databases is their fixed schema which defines the structure of the data. Semi-structured documents, however, have only loose constraints on the data structure (20), which cannot be handled efficiently by relational databases (21). As all standards for model encoding are semi-structured, relational databases are not the best choice for efficient storage.

NoSQL approaches, together with semantic web applications, more recently gained popularity in the life sciences (22), e.g. as Key-Value Stores, BigTable (23), document databases, triple stores or graph databases (24). We chose the graph database Neo4J (25). It represents data in terms of nodes, edges and attributes. Nodes are connected via directed edges (relations) of certain types. Both nodes and edges can then hold attributes. The Neo4J architecture follows the fundamental properties of databases, i.e. the ACID principles (atomicity, consistency, isolation and durability).

### Incorporated data domains

Several types of data are relevant for a meaningful description of computational models in biology (7, 18, 26). Specifically Knüpfer *et al.* (7) distinguish data for the extrinsic and intrinsic description of model function, behavior and structure. Many of these aspects have already been described in standard formats, including model structure (27–29), simulation descriptions (30), simulation results (31) and semantic annotations (32, 33, 34). In this work, we focus on the data requested by two Minimum Information Guidelines: MIRIAM for requested information about models and MIASE, the Minimum Information About a Simulation Experiment (14), for requested information about simulation setups.

The development of standards is a continuous process, and their uptake by software tools and users progresses at varying speed. For example, while many journals today recommend, or require, the provision of model code during submission (e.g. in SBML), there is no such recommendation to submit also a graphical representation in the Systems Biology Graphical Notations (SBGN) (29), nor to submit the simulation description (in SED-ML). Some formats are specified, but so far only used by a small number of software tools, e.g. the Systems Biology Result Markup Language (SBRML) (31). However, repeated calls for model reproducibility have been published in the past years (13, 14). The ongoing development of standards fosters both the submission of model-related data to model repositories such as BioModels Database and the distribution of archives such as the Research Objects (35) or the recently launched COMBINE Archive (36). In the following, we will only consider types of data that have been formally specified and for which curated data is available. These are basically the model code, simulation descriptions, semantic annotations and cross-references, and the mathematical characterization of models (18).

#### Model code in public repositories

Modelers predominantly implement their models in native programming languages, most commonly C or C++; script languages such as MATLAB or Python and using graphical representations. Program code and scripts, in general, are hard to understand and share. An XML representation reduces the obstacles to sharing data among diverse applications by providing a common format for expressing data structure and content (37). XML formats for the standardized representation of models are SBML, CellML or NeuroML (38). They all focus on the encoding of the models’ structure, for example the interactions in a pathway, and describe sets of entities and the processes between them. Hucka *et al.* (27) highlight the advantages of markup languages, in this case SBML, for model representations: model definition becomes straight forward, and a tool chain is available. BioModels Database, for example, guarantees persistence and long-term availability of 548 curated models (Release 28 of BioModels Database as of 16 September 2014) and several thousands of automatically generated pathway models which have been generated from the KEGG database (39). Many simulation tools read and write models in SBML (6).

#### Simulation descriptions

The ability to represent increasingly complex biological phenomena requires models to be instantiated using different initial conditions and parameters, and these conditions must be formally described together with the model itself (40). For example, in pharmacometrics, the calculation of a parameterization of an individualized model is itself a complex procedure that requires the development of further standards (41). To ensure the reproducibility of simulation results, the SED-ML (19) is an XML-based format that encodes the necessary information to reproduce a particular result. SED-ML Level 1 Version 1 (30) enables the reproduction of time course simulations. The more recent Level 1 Version 2 covers a broader range of experiments, including pulse experiments and parameter scans (42). BioModels Database and the CellML Model Repository provide SED-ML files for selected models in their repositories. Surprisingly though these SED-ML files are not linked with the models inside the databases. Consequently, the information about applicable simulation experiments for a model is not computationally accessible. This information is, however, desired by researchers who wish to define generic experimental setups, so-called virtual experiments (40), and link these to sets of models for comparison, validation and functional curation (43). Hence, simulation setups are a kind of meta-data that are relevant for database design decisions.

#### Semantic annotations and cross-references

Semantic annotations link model entities to terms in bio-ontologies. Ontologies, in general, are defined as specifications of a conceptualization (44). Bio-ontologies, e.g. GO, that are ontologies with a focus on biological terms. Many cross-references between ontologies are provided in BioPortal (45). Both SBML and CellML use bio-ontologies to enrich model descriptions with semantic annotations, using Resource Description Framework (RDF) triples (46). In BioModels Database, models carry between 3 and 800 annotations, but on average 71 annotations, per model (47). For example, an annotation could be added to the SBML species X, linking it to the ontology term ATP in ChEBI (48) (ID CHEBI:15422). So-called qualifiers specify the relation between entity and ontology term (49). A model entity could be ATP or have a part ATP. The sum of semantic annotations in a model describes its biological and mathematical background.

### Database design and data import

The following section describes the structure of our database. For demonstration purposes, we use one of the early Tyson models on cell division (50). This model is fairly small, it is available in SBML [BioModels Database (http://www.ebi.ac.uk/biomodels-main/BIOMD0000000005)] and CellML [CellML Model Repository (http://models.cellml.org/exposure/9bff394be3ade829feed94151b3d68b3/tyson_1991.cellml/view)] format.

A so-called document root node is created for each data item. It is the entry point to the database. Attached to this node can be a model code (e.g. an SBML node) or model-related data item (e.g. a SED-ML node). The entry point for each ontology is a so-called ontology root node.

**Figure 1.**
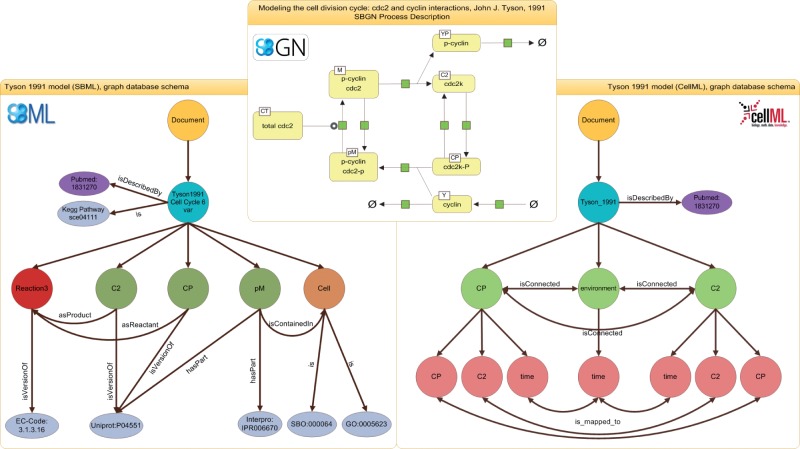
Representations of the Tyson 1991 model. The SBGN (top) representation shows the process description for the Tyson 1991 model. The representation of the SBML model inside the graph database is shown on the left, the representation of the CellML model is shown on the right. The document node is colored in yellow, model nodes in blue, annotation nodes in silver and publication nodes in purple. For the SBML representation, reaction nodes are red, species nodes are dark green and compartment nodes are brown. For the CellML representation, component nodes are light green and variables are light red. The figure shows only an excerpt of the model representation, for example many nodes and edges are omitted in favor for readability.

More specifically, SBML models (Figure 1, left) are represented by a model node which stores the model’s name (in cyan color) and identifier. Attached to the model node are annotation nodes, including the reference publication (purple and gray). The model node is also connected to reaction, species and compartment nodes to reflect the underlying structures in the biological network. The example in Figure 1 shows a subset of nodes and edges for the Tyson model. All information about these nodes is directly extracted from the model’s SBML representation. The figure displays three species nodes (in green), one reaction node (in red) and one compartment node (in orange). The edge between the species node pM (a complex of phosphorylated Cyclin and phosphorylated cdc2) and the compartment node Cell represents the fact that the species pM is located in the compartment Cell. The qualifiers in the SBML model (13, 34) allow us to incorporate further information on the type of relation between an entity and an ontology concept, e.g. that pM is linked to Cell via the relation isContainedIn. Further model entities are stored analogously, i.e. encoded parameters, events and other SBML concepts. For example, for each global parameter a node is created and attached to the model node. The same procedure holds for functions. Only unit declarations are currently omitted, they may be implemented in future versions of the graph database, e.g. for applications such as model merging. Finally, the semantic annotations are extracted from the SBML model and stored. The use of graph databases makes this mapping intuitive: nodes representing some model entity are linked to nodes representing a particular term in a bio-ontology. The edge specifies that relation. An additional node is created and connected each time a new URI is detected during model import. For our example, the species node pM is related via hasPart to the InterPro term Diphthine synthase (in gray). Taken together, the sum of extracted information provides a detailed representation of the models’ network structure and all annotations.

*CellML models* represent networks of connections between so-called components. A component contains variables and mathematical relationships that manipulate those variables (28). This is a different, more abstract approach to representing reaction networks, and one of the reasons why an integrated storage of SBML and CellML models on the XML level is so difficult (51). Examples for CellML components are physical compartments, events, species or other convenient modeling abstractions. As for SBML models, the entry point is a document node that is connected to a model node and serves as an anchor for the component nodes. Each model entity can be related to a semantic annotation. Figure 1 (right) shows the CellML representation of the above-mentioned Tyson model. Attached to the model node are the component nodes, for example C2, Cp or environment. Each component holds a number of variables. These variables are mapped to corresponding variables of connected components, e.g. the variable time in component node C2 is connected to the variable time in the environment node. Please note here that the model node links to the identical publication node as the SBML model. If existing, annotations are extracted from the CellML model and mapped to the database using the same URI scheme as with SBML models. Although CellML models today are only sparsely annotated, several projects work toward fully annotated CellML models (33, 52, 53). Our database is updated accordingly.

**Figure 2.**
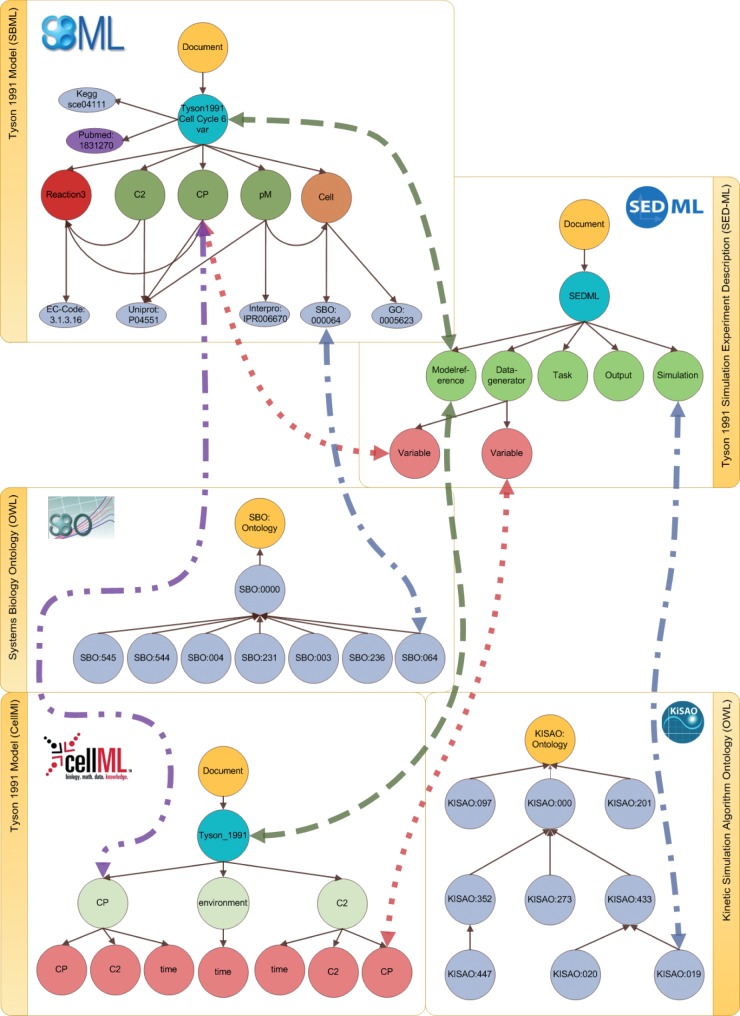
Linking models, simulation descriptions and ontologies. The linking between different data domains is shown: simulation experiment descriptions and models (dashed line); defined observation variables and model entities (dotted line); annotated model entities and simulation experiment descriptions (dashed-dotted line) and model entities of different representation formats (double dotted-dashed line). The SBO example is explained in detail in the Implementation section. The references to the simulation algorithm within a simulation experiment description are mapped to the corresponding entity in KiSAO. All annotations referring to GO are mapped, but not shown in the figure.

*SED-ML descriptions* specify simulation setups for models. They thereby link models, simulation algorithms and output definitions (plots). A SED-ML description also explicitly declares the observed variables. In our design, the SEDML node serves as the anchor for an experiment. The Modelreference node links the experiment to all Model nodes used in the simulation. Figure 2 exemplifies how a model reference links one SED-ML description to an SBML and a CellML model. Algorithms used for simulation are described by concepts from the Kinetic Simulation Algorithm Ontology (KiSAO) (54), which is one of the bio-ontologies that we import into our database. A subset of KiSAO terms is depicted in Figure 2.

We incorporate the concepts of frequently used *bio-ontologies* to be able to query the information hidden in the semantic annotations of in model representations and simulation descriptions. For example, model entities are mostly annotated with concepts from SBO, GO, ChEBI (47); simulation descriptions contain links to simulation algorithms in KiSAO. Most bio-ontologies are available in the Web Ontology Language (OWL), which is a standard format for the representation of semantic information on the web. We parse these ontologies and add all concepts and relations as nodes and edges, respectively. Cross-references between concepts of different ontologies are currently not mapped to the database.

Table 1 summarizes the data types, number and size of the documents in our database. The integration of further data resources is possible using import tools. For example, we offer a generic importer for ontologies encoded in OWL. However, the links between the incorporated ontology concepts, models and model-related data need to be defined manually. Adding data encoded in SBRML (31) or NuML (http://code.google.com/p/numl/) would require additional importers and again a manual post-processing.

### Linking model-related data

The main advantage of the previously described concept is its possibility to define flexible links between the data domains. In concordance with previous considerations (16, 18), we incorporate the following types of links:

- links between annotations (in SBML, CellML and SED-ML) and ontology concepts,
- links between models (in SBML or CellML format) and SED-ML,
- links between model entities and SED-ML variables and
- links between model entities from different model representation formats.

Figure 2 depicts all links for the Tyson model.

The database contains the SBML and CellML representations of this model. Both representations are outlined on the left-hand side of the figure. The first type of link is between model entities and ontology concepts (a). Here, we only consider existing annotations. For each annotation in a model we add an explicit link to the data entry in the referenced bio-ontology. For example, based on the SBO annotation in the SBML model we build an additional edge between the node representing that annotation in SBML and the entry in SBO itself. Each concept (from an ontology) is only stored once but can be referred to by multiple model entities.

Another type of link is that between a model and a simulation description (b). When importing a SED-ML file into the database, we resolve the model references. We then check if those models are contained in our database. If this is the case, then additional edges are introduced for each model reference, between one model node and one SED-ML Model reference node at a time. In the example in Figure 2, the original SED-ML file contained two model references, pointing to the Tyson 1991 model in SBML and CellML format, respectively. Thus, we add two edges.

Furthermore, the variables of a DataGenerator in a SED-ML file may point to a specific entity in the referenced model. This pointer is used to identify the entity under observation, or for pre-processing before simulation. Although we do not store the specific processing of a model entity, we keep the information if a model entity is part of a simulation. A third type of link thus relates DataGenerator nodes with model entities (c) when a SED-ML file is imported. Also, we flag species that are altered during SED-ML pre-processing (e.g. if the concentration of a species is changed).

The links (a)–(c) can be inferred from information encoded in the models. Therefore, we regard them explicit links. In addition, we determine implicit links between models of different representation formats (d). As we showed earlier, two models may link to the same publication (Pubmed:1831270 in Figure 2). If two models share a publication, the systems can infer implicit links between those entities that are equally named. Entities with similar names (e.g. in terms of Levenshtein Distance or stemming) also have a high probability of being identical. The confidence can be increased further if the entities’ annotations match. Figure 2 shows the explicit connection of the entities C2 in the SBML and C2 in the CellML model. Both entities are linked because they have the same name, and they stem from the same reference publication. Please be aware that linking two models based on the ideas described above does not necessarily mean these models are equal. It only means that they are similar. If and when models can be considered equal is an ongoing discussion that is not part of this work.

## Discussion

### Advantages of implementing a graph-based concept

The main advantages of a graph-based concept for model storage are easy integration of heterogeneous resources, extensibility with further data resources and improved model search.

Currently, models and model-related data are only sparsely linked in the, predominantly relational, model repositories. Relational databases store data in tables and use the concept of primary and foreign keys, making them a strong tool for the storage of structured, homogenous data. On the contrary, they do not perform well on highly connected, semi-structured and heterogeneous XML data. In a graph database, the integration of heterogeneous resources is straight forward. The concept of edges allows arbitrary connections to be defined by the creators of the database at any time. Particularly helpful for later model comparison are edges that connect nodes across model representation formats. For example, our database contains two representations of Tyson’s 1991 cell cycle model, in SBML and in CellML, respectively. The two models are connected via an edge between the model nodes. This link now becomes exploitable, because both model nodes share one publication node (PubMed:1831270). It is also useful to represent relations between a model and a simulation setup. Storing this information in the graph database allows modelers to quickly retrieve all models associated with a simulation experiment, and ‘vice versa’. For example, our graph database contains the information that there exists a SED-ML file which simulates and observes the change in concentration in CP in both encodings of the Tyson 1991 model (SBML and CellML) and then compares the simulation outcome (Figure 2). Finally, our database establishes links from model annotations into bio-ontologies. For example, the SBML model in Figure 2 contains the entity Cell which is annotated with a term from SBO. We can thus easily retrieve all models that are annotated with a particular ontology term. This is, for example, helpful in the classification of models as we show in Alm *et al.* (47).

Our graph database is schema optional. Thus, new data resources can efficiently be integrated and the database easily be extended. We plan to integrate links to result data (in NuML format) and to wet lab descriptions once these exist in standard format. Data in NuML format could be linked to model entities, for example, when storing different parameterization of a particular model to formally describe its variants.

As current repositories do not represent the structure of a model, they cannot answer questions such as ‘Which model in the database contains the species that modifies most reactions?’. To identify a species as the modifier of a reaction, this information must exist in the database. Figure 1 shows how we keep the information on the model structure: for each reaction in the model we map all reactants, modifier and products. Participants in the reactions are furthermore linked to the bio-ontologies, to simulation setups and linked across the SBML and CellML representations. Building on this structured representation, a query can now add restrictions on the reaction network, e.g. formulating the following two conditions: (1) the species should only serve as a modifier in any of the model’s reactions and (2) only the topmost species per model should be considered. For this specific example, our graph database retrieves the model ‘Schaber2012—Hog pathway in yeast’ (Originally from BioModels Database, http://www.ebi.ac.uk/biomodels-main/BIOMD0000000429), because the species Hog1PPActive occurs in 10 reactions and only acts as a modifier (Query 1). (The following examples are based on BioModels Release 25, curated branch. A list of CellML models used in the following examples is available as Supplementary Material)

MATCH (species:SBML_SPECIES)-[isMod:IS_MODIFIER] - >()

WHERE NOT((species)-[:IS_REACTANT] - >() OR (species)-[:IS_PRODUCT] - >())

WITH species, count(isMod) AS numOfMod ORDER BY numOfMod DESC LIMIT 1

MATCH species-[:BELONGS_TO]->model

WHERE (model:SBML_MODEL)

RETURN model.NAME AS Model, species.NAME as Species, numOfMod

Query 1: Return the model with the most species acting only as a modifier.

Result 1: The model “Schaber2012 - Hog pathway in yeast” having the species Hog1PPActive which is acting as a modifier in 10 reactions.

Graph databases offer further exciting applications, including the structure-based comparison of models. Combinations of nodes and edges form sub-networks which can for the first time be compared with each other using graph matching techniques. Once specific algorithms to map sub-models and identify suitable interfaces for automatized model coupling are in place, it will be possible to integrate them with our ranked retrieval system (10). The ‘Materials and methods’ section contains further examples.

### Exploiting links to associated virtual experiments

‘Which simulation experiments in the repository investigate the change of concentration in “m-phase inducer phosphatase”?’ To answer this question, it is not sufficient to query the model only. The retrieval system must also incorporate information about, and links to, simulation experiments. Sometimes open repositories supplement models with SED-ML files, enabling users to reproduce one or more figures of the reference publication. However, current model repositories do not explicitly store the link between model and simulation description. Consequently, one cannot retrieve relations of that kind. For example, Novak’s 1997 cell cycle model can be run with at least two different setups, either reproducing Figure 2a or b of Novak and Tyson (55). Our implementation keeps the links between simulation setups and models and thus knows which experiments are applicable to which models. Query 2 is an example for the retrieval of all known simulations for a model. The links between SED-ML elements and KiSAO allow us to define restrictions on the SED-ML files we want to consider in a search result, e.g. to retrieve only models that can actually be simulated with a given simulation algorithm. With our system, the query ‘Which CellML encoded models can be simulated using a Livermore Solver?’ can be answered (Query 3). One can also imagine to restrict results to changes in concentrations of a certain parameter. Finally, SED-ML descriptions may be defined as templates for virtual experiments. Virtual experiments are *in silico* assays of a model’s behavioral repertoire, both in declaring what a model should do and verifying what it actually does (40). Such simulation descriptions are per definition applicable to classes of models, enabling the clustering of models by type of experiment that they reproduce correctly.

MATCH (m:SBML_MODEL)-[:REFERENCES_SIMULATION_MODEL]-ref-[:BELONGS_TO*2]->(sed:DOCUMENT)

WHERE m.NAME=’Novak1997 - Cell Cycle’

RETURN m.NAME AS Model, m.ID as ModelID, ref.MODELSOURCE as ModelSource, sed.FILENAME as SEDMLFile

Query 2: Return all simulations that can be applied to the model ”Novak1997 - Cell Cycle”

Result 2: The requested model can be run by two simulations, reproducing Figure 2a and 2b by [42]

MATCH (sed:DOCUMENT)<-[:BELONGS_TO*2]-(sim:SEDML_SIMULATION)-[:SIMULATES]->

(ref:SEDML_MODELREFERENCE)-[:REFERENCES_SIMULATION_MODEL]->m

WHERE

(sim.SIMKISAO=’KISAO:0000019’) AND  filter(lable  in  labels(m) where lable =’CELLML_MODEL’)

RETURN m.NAME, sed.FILENAME

Query 3: Return only CellML models that can be simulated using a Livermore Solver (KISAO:0000019)

Result 3: The CellML encoded ”Tyson 1991” model and the corresponding SED-ML file.

START res=node:annotationIndex(’RESOURCETEXT:(m-phase inducer phosphatase)’)

MATCH res<-[rel:is]-(a:ANNOTATION)-->(s:SBML_SPECIES)<-[:OBSERVES]-o-[:BELONGS_TO*]->(doc:DOCUMENT)

WITH doc,res,s,o

MATCH ()<-[:IS_MODIFIER]-s-[:BELONGS_TO]->m

RETURN DISTINCT doc.FILENAME AS SEDML, collect(distinctm.NAME) AS Model,

collect(distinct res.URI) AS Resource, collect(distinct s.NAME) AS Species, collect(distinct o.TARGET) AS Target

Query 4: Return simulation descriptions observing a particular species that plays the role of a modifier or reaction, respectively. The observed species should be annotated as “m-phase inducer phosphatase” using the qualifier *is*.

**Figure 3.**
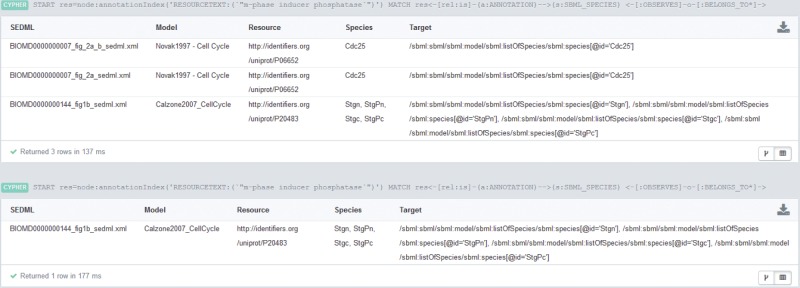
Results for Query BM3. The query output at the top of the figure restricts the species role to ‘modifier’. Three SED-ML files match. The first and second files belong to the same model and both observe the species *Cdc25*. The third query result is a SED-ML file observing four different species. The query output at the bottom of the figure shows the result of a similar query. Here the species must act as ‘reactants’. Only one SED-ML file is retrieved, namely the third result of the top query. All retrieved species (declared as observed by a SED-ML file) are annotated with a UniProt ID. The annotation is either *P06652*, the protein *Cdc25* in yeast, or *P20483*, the protein *Stg* (*Cdc25*) in the fruit fly. Simulation files for CellML files are not retrieved, because CellML files are not yet fully annotated. If the CellML version of the Novak 1997 model had annotations corresponding to ‘m-phase inducer phosphatase’, the database would have also returned the simulation description for that model.

Result 4: The result is shown and explained in Figure 3.

Strikingly, it is also possible to derive information from the graph database by combining the different data sets. Query 4 shows such a complex example. It combines index and structure information and spans data sets of ontology, models and simulation experiments. It retrieves a simulation experiment description and corresponding models where a species is marked for observation by the simulation description. Additionally, the observed species must be annotated with a resource that is related to the phrase ‘m-phase inducer phosphatase’ and the species must play the role of a modifier. The result is shown in Figure 3. To our knowledge this is the first time a system can answer queries spanned over different data sets and combining them with an index look-up.

### Statistics

Our graph database can also provide interesting statistics about models. For example, we can identify the term SBO:0000009 (kinetic constant) as the most frequently used annotation in BioModels Database (Query 5). We can also compute the number of annotations using SBO:0000009 or one of its 125 children (Query 6). Finally, the system can derive statistical values. For example, the average number of annotations per model, as well as the minimum, maximum and the standard derivation, can be computed for the set of SBML and CellML models available from BioModels Database and the CellML Model Repository, respectively (Query 7).

MATCH (r:RESOURCE)-[qualifier:BELONGS_TO]->()

WITH r, count(qualifier) AS AnnotationCount ORDER BY AnnotationCount DESC LIMIT 3

RETURN r.URI as Annotation, AnnotationCount

Query 5: What are the top-most three annotations used?

Result 5: Top three annotations used are SBO:0000009 (kinetic constant, used 1127 times), SBO:0000252 (polypeptide chain, used 509 times), GO:0043241 (protein complex disassembly, used 484 times)

MATCH ()-[rel]->(res:RESOURCE)-[:IS_ONTOLOGY_ENTRY]-c-[:isA*0..]->s

WHERE s.id=”SBO_0000009”

RETURN count(rel)

Query 6: How many annotations point to the term SBO:0000009 (kinetic constant) or one of its children?

Result 6: 3373 annotations pointing to SBO:0000009 or one of its children (e. g. half-life, diffusion coefficient or bimolecular rate constant), 1127 of them point directly to SBO:0000009.

MATCH (m:SBML_MODEL)<-[:BELONGS_TO*1..2]-(a:ANNOTATION)<-[:BELONGS_TO]-(r:RESOURCE)

WITH m as Model, count(r) AS NumberOfAnnotation

RETURN max(NumberOfAnnotation), min(NumberOfAnnotation), avg(NumberOfAnnotation), stdev(NumberOfAnnotation)

Query 7: What is the minimum, maximum and average number of annotations per model?

Result 7: A model has a maximum of 800, a minimum of three and an average of 71 annotations.

### Comparison with other approaches

#### RDF-triple-stores and SPARQL

Semantic Systems Biology has been termed the new field of research that aims to improve formal knowledge representation of computational models to enhance construction, comparison, validation, or retrieval (9). Several projects convert model representations into semantically enriched formats to compare models and to improve the integration with knowledge in bio-ontologies (56, 57). In general, the idea is to transform all data into RDF representations, store the RDF triples into databases and provide SPARQL (http://www.w3.org/TR/rdf-sparql-query/) endpoints to access the triples. For example, SBML models in BioModels Database can be converted into OWL or RDF representations, using straight forward to more complex transformation methods (51, 56–58). SPARQL has become the de-facto query language for the Semantic Web community and is also used in the domain of computational biology, e.g. Bio2RDF (http://bio2rdf.org/) (59) or recently the BioModels Database SPARQL endpoint (60). Although many formats can be transformed to RDF, an RDF representation is not available for all data we included (i.e. SED-ML). An in-depth comparison of graph-databases with RDF triple-stores (and associated query languages) is not in the scope of this article. The major reason for us to use a graph database was their support for graph algorithms. Triple-stores and SPARQL are tailored toward sub-graph retrieval. However, common graph algorithms such as Dijkstra’s algorithm (shortest path), directed path traversing, spanning trees or sophisticated graph matching patterns are hardly applicable on RDF triple-stores (61). We argue that the graph structure and thus graph algorithms will become more important in the domain of computational biology. We already used our graph database to extract characteristic features for sets of thematically similar models (e.g. cell cycle or NF- κ B) (47).

#### BioModels Database

**Figure 4.**
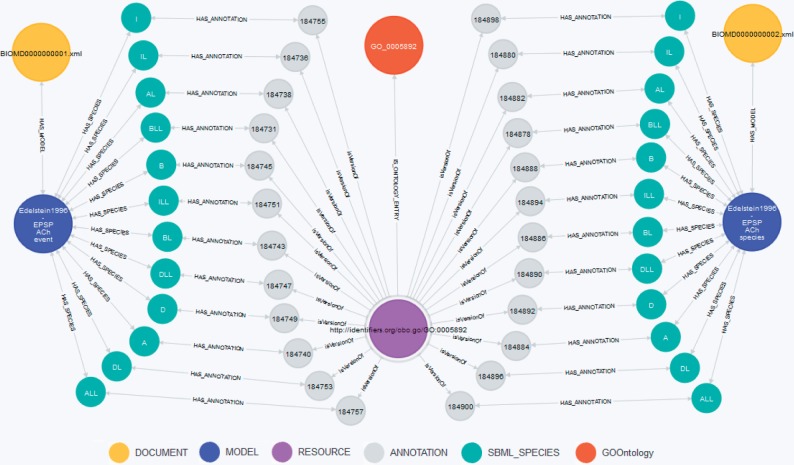
Visualization of Query BM3. The centered node (purple) is the requested annotation *GO:0005892* (acetylcholine-gated channel complex). This node is connected to the orange node representing the GO term within the GO. The green nodes represent the species linked to the *GO:0005892* annotation, the blue nodes represent the models ‘Edelstein1996—EPSP ACh event’ (BIOMD0000000001) and ‘Edelstein1996—EPSP ACh species’ (BIOMD0000000002). Documents are displayed as yellow nodes. Nodes in gray represent annotation containers as stated in the SBML specification (4) and do not carry any meaning themselves.

Recently, the European Bioinformatics Institute in Hinxton, UK, announced a SPARQL endpoint for BioModels Database. All SBML-encoded models in BioModels Database were converted into RDF representations and added to the EBI RDF Platform (60). For comparison of our concept against BioModels Database’s approach, we translated the query examples from the EBI web page (http://www.ebi.ac.uk/rdf/services/biomodels/) into queries against our graph database. The executed queries and retrieved results are shown in the following three listings. Additionally, Figure 4 shows a visualization for Query BM3.

MATCH (m:SBML_MODEL)-->(s:SBML_SPECIES)

WHERE (m.ID=”BIOMD0000000001”)

RETURN m AS Model, collect(s.ID) as SpeciesID, collect(s.NAME) as SpeciesName

Query BM1: From model BIOMD0000000001, list all species identifiers and names

Result BM1: 12 species IDs (ALL, I, DL, ILL, D, DLL, B, BL, A, AL, IL, BL) and names (ActiveACh2, Intermediate,  …)

MATCH (r:RESOURCE)-->()-[:BELONGS_TO]->(element)-->(m:SBML_MODEL)

WHERE m.ID=”BIOMD0000000001”

RETURN element.ID AS Element, LABELS(element) AS ElmentType, collect(r.URI) AS ElementAnnotation

Query BM2: Get element annotations of the model BIOMD0000000001

Result BM2: 104 annotations for 65 distinct elements, for example species ALL is annotated with IPR002394, GO:0005892 and SBO:0000297

MATCH (r:RESOURCE)<-[rel]-()-->e-[:BELONGS_TO]->(m:SBML_MODEL)

WHERE r.URI=∼”.*GO.*0005892”

RETURN m.ID AS ModelID, collect(e.ID) AS ElementIDs, type(rel) AS Qualifier, r.URI as URI

Query BM3: All model elements with annotations to acetylcholine-gated channel complex

Result BM3: From each model (BIOMD0000000001 and BIOMD0000000002) the same 12 species IDs are returned (ALL, I, DL, ILL, D, DLL, B, BL, A, AL, IL, BL), all are qualified with *isVersionOf*. A graphical representation is shown in Figure 4.

We could easily apply all queries and reproduce the results. Specifically, query BM3 asks for models annotated with the ‘acetylcholine-gated channel complex’. Due to the missing index support for this RDF store, the user must first manually look up and transform this annotation term into a URL, and paste that into the query. Our system, in the contrary, is able to retrieve this information automatically by a simple index-based query. A detailed example for the automatic conversion of an annotation is given in the aforementioned Query 4 or in Query M6 in the Materials and methods section.

#### COMBINE Archive

The types of meta-data considered in this work are also agreed upon by an effort called the COMBINE archive (18), which aims at publishing extractable archive files that then contain all files necessary to reproduce a scientific modeling result in the life sciences.

The COMBINE archive is well suited for the export and exchange of research results. Our graph database rather offers a solution for the management of those files. For example, the CombineArchive Toolkit (62) may query our database, collect all necessary information and automatically create an Open Modeling EXchange format file (http://co.mbine.org/specifications/omex.version-1).

### Conclusion

Open model repositories are frequently queried for computational models describing particular aspects of biological systems. However, their storage concepts are restricted and not all data contained inside the repositories are incorporated into the search process.

The system described in this article incorporates and links knowledge that is in principle already available in public repositories, but not yet utilized. The knowledge is encoded in meta-data, in particular links to simulation experiments and semantic annotations with terms from bio-ontologies. The key to using this knowledge in model management tasks is its explicit linking and indexing in the database. We demonstrate how relevant meta-data can be stored in a graph-database using the example of Neo4J. We furthermore exemplify how this meta-data can subsequently improve model retrieval and thus model reuse.

Our concept is easy to adapt and implement. An interface to test and query the database described in this article is available (https://sems.uni-rostock.de/projects/masymos/). In addition, a web API (https://sems.uni-rostock.de/projects/morre/) designed to search SBML, CellML and SED-ML files is available for testing. A prototype implementation is running as a search service on an instance of the Physiome Model Repository, which is the backend of the CellML Model Repository (http://staging.physiomeproject.org/).

## Materials and methods

### Mapping XML-encoded models and model-related data to the graph database

The entry point for each model import is a document node. It links to a model node via the directed edge hasModel. The model node has a model name and relations (i.e. edges) to nodes that represent model entities. In the case of SBML these entities include species, compartments or reactions. For example, a model’s species is represented by its own node. Additionally, an edge from the model node to the species node is created and named hasSpecies. Nodes for each reaction and compartment are created and connected with hasReaction and hasComartment, respectively. Moreover, relations of model elements are mapped to the graph database, i.e. a species node is connected to a compartment node with isContainedIn. To ensure an easy traversal upwards, a connection is created from each node of the stored model that points to the parent of the current node. The corresponding edges are named belongsTo. Furthermore, it is possible to attach an annotation to each model entity, describing the particular entity in more detail. All such annotations are stored to the database and indexed. The textual descriptions of terms in ontologies such as GO or ChEBI are retrieved from the according web pages, indexed and then processed. This index is afterwards used for ranked model retrieval as described in Henkel *et al.* (10). Attached to every node is a so-called label that names the type of node, e.g. species, compartment or annotation. Labels are indexed and allow to select all nodes of a specific type.

### Implementation

**Figure 5.**
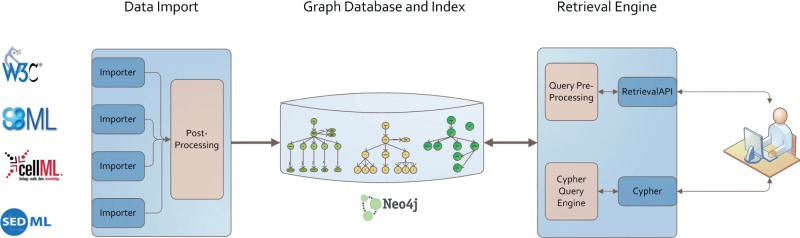
Architecture of our graph database. Data from different models, simulation descriptions or ontologies are imported using format-dependent importers. Each import undergoes a post processing afterwards. The stored graph and index structures are available via two retrieval interfaces: Cypher and an adaption of Henkel *et al.* (10). Both are based on RestAPIs. The data itself are stored in a Neo4J graph database.

We implemented the graph-based storage according to the architecture depicted in Figure 5. The Neo4J (http://www.neo4j.org/) database stores model files, simulation descriptions and model-related information in a graph manner. The retrieval engine is based on the ranked retrieval described in Henkel *et al.* (10). It allows users to access the data in the database, and retrieve ranked lists of results for their text queries. Queries are resolved using the Lucene framework (http://lucene.apache.org/core/), and ranked based on predefined similarity features. The data import pushes different data formats, including model code, simulation experiment descriptions and ontologies, into the graph database. Afterwards a post-process takes care of linking the added data of different domains.

*Models and Simulation Descriptions* are added to the database using format-dependent importers. Each supported format has its specification. Consequently, importers were implemented for SBML [based on jSBML (63)], for CellML and for SED-ML [based on jlibsedml (19)]. All importers share a common interface which maps the model and simulation files onto a graph structure. The import keeps the models’ semantic information and all content that is relevant for later model querying, retrieval and display.

*Bio-ontologies* available in OWL can also be imported using the owl-API (http://owlapi.sourceforge.net/) and the JFact (http://jfact.sourceforge.net/) reasoner. However, after adding an ontology to the database a post-processing is required to link model or simulation description entities to the newly added Ontology concepts. This post-processing is part of the linking process.

*Linking models and simulations* is done using the graph query and data modeling language Cypher (http://www.neo4j.org/learn/cypher) (21), which is shipped along with Neo4J. The following query shows the command to link SBO annotations of models to the corresponding concepts of the SBO using Cypher.

MATCH (res:RESOURCE), (sbo:SBOOntology)

WHERE (res.URI =∼ ”.*SBO.*”) AND (RIGHT(res.URI, 7) = RIGHT(sbo.id, 7))

CREATE res-[link:IS_ONTOLOGY_ENTRY]->sbo

RETURN count(link);

Query P1: Select, match and link SBO annotations extracted from models with corresponding concepts from the SBO.

Result P1: The number of created links.

The *MATCH* clause selects every node that is labeled with the term ‘RESOURCE’ and ‘SBOOntology’ into the variable res and sbo, respectively. The *WHERE* clause restricts the selection to only those nodes satisfying the following constraints. In this case, the attribute URI of a resource node must contain the string ‘SBO’ and the last seven digits must correspond to the last seven digits of a node id out of the selected SBO concepts. This pairs all SBO annotations used in a stored model with the corresponding entry within the Systems Biology Ontology. For the selected pairs of nodes, the *CREATE* clause adds a new directed edge to the graph connecting both nodes. The label of the selected edge is IS_ONTOLOGY_ENTRY. Finally, the *RETURN* clause counts the number of edges created by this command and returns it to the user. A similar procedure applies to other bio-ontologies.

### Supported types of queries

The Cypher Query Language provides direct access to the data in our graph database. Cypher is the declarative language to pose queries against graph structures, similarly to SQL for relational databases. Our system supports standard queries such as data look-ups, filtering and aggregation. In addition, more complex queries regarding the model’s structure can be posed.

#### Look-up and filtering

Examples for database look-ups and filtering are shown in Query M1 and Query M2. The *MATCH* clause uses a build-in index to retrieve all nodes labeled as CellML model (Query M1) whereas the *WHERE* clause restricts the nodes to the ones matching the given name (Query M2). The result of the first query is a list of 841 CellML models, while the second query returns only the Tyson 1991 model.

MATCH (m:CELLML_MODEL)

RETURN m

Query M1: Database look-up. Return all CellML models

Result M1: List of 841 models

MATCH (m:CELLML_MODEL)

WHERE m.NAME=’tyson_1991’

RETURN m

Query M2: Database look-up and filtering. Return CellML models with the name “tyson_1991”

Result M2: A model node containing the attribute NAME: “tyson_1991”

#### Graph matching

Query M3 shows how graph structures can be queried. In this example, all components from the Tyson 1991 model are selected. Eight component names are returned, as denoted in the *RETURN* clause.

MATCH (m:CELLML_MODEL)-->(c:CELLMLCOMPONENT)

WHERE m.NAME=’tyson_1991’

RETURN c.NAME

Query M3: Database graph structure query. Select the aforementioned Tyson model and return all its components.

Result M3: The components YP, Y, M, pM, CP, C2, environment and reaction_constants.

#### Aggregation

In SQL, aggregation functions such as count() or sum() group values from multiple rows into a single value. Query M4 shows how to define a query that counts the number of species for each model in the graph database. The *MATCH* clause selects the Tyson 1991 model, all connected components and variables. The *RETURN* clause counts and returns the number of variables for this model. Further examples of aggregation queries are given in Table 1 in the Results section.

MATCH (m:CELLML_MODEL)-->(c:CELLMLCOMPONENT)-->(v:CELLMLVARIABLE)

WHERE m.NAME=’tyson_1991’

RETURN count(v)

Query M4: Database aggregation query. Count the number of variables contained by any component of the aforementioned Tyson model

Result M4: This model has 68 variables.

#### Statistics

Cypher also supports statistical queries. Query M5, for example, returns the minimum, maximum and average number of variables attached to components in CellML models. To provide these statistic values, elements (in this case the CellML components) are selected and bound to an aggregation value using the *WITH* clause.

MATCH (m:CELLML_MODEL)-->(c:CELLMLCOMPONENT)-->(v:CELLMLVARIABLE)

WITH c as component, count(v) as NumOfVar

RETURN min(NumOfVar), max(NumOfVar), avg(NumOfVar), stdev(NumOfVar)

Query M5: Statistics query. Retrieve minimum, maximum average and standard derivation of for the number of variables attached to a component.

Result M5: A minimum of one and a maximum of 431 variables are attached to a component of a CellML model. On average each component has 9.64 variables attached with a standard derivation of almost 16.

#### Index support

Finally, Query M6 uses an index to retrieve nodes matching a given pattern. The indexed annotations are queried for the term ‘m-phase inducer phosphatase’ using the *START* clause.

START res=node:annotationIndex(’RESOURCETEXT:(m-phase inducer phosphatase)’)

RETURN res

Query M6: Database index query. Retrieve all annotations containing the phrase “m-phase inducer phosphatase”

Result M6: A set of seven resources (InterPro IPR000751; Enzyme Commission number 3.1.3.48; and UniProt: P30311, P23748, P20483, P06652, P30304)

### Database scaling

Büchel *et al.* (39) describe how to build computational models from biochemical pathway maps. The path2models (http://code.google.com/p/path2models/) project resulted in more than 140.000 SBML models of a total size of 70 GB. We used this data set to challenge the database’s performance on an average office system (Intel Core 2 Quad @ 2.66GHz CPU, 8 GB RAM, Windows7 64 Bit). The database was created in 20 h and 40 min, thus every model required 531 ms on average. Although importing the path2models project, 45.5 million nodes and 492 million relationships were created; the database size is approximately 83 GB, including the indices.

## Author contributions

R.H. and D.W. designed the project and wrote the paper. R.H. implemented the storage system. O.W. reviewed paper drafts and supervised the project.

## Supplementary data

Supplementary data are available at *Database* Online.

Supplementary Data
