# Supplementary material for: Combining computational models, semantic annotations and simulation experiments in a graph database
Source: Database (Oxford). 2015 Mar 8;2015:bau130. doi: 10.1093/database/bau130 (PMC4352687; doi:10.1093/database/bau130)
Supplement: Supplementary Data [file supp_2015_bau130_index.html]

Combining computational models, semantic annotations and simulation experiments in a graph database — Supplementary Data 

# Combining computational models, semantic annotations and simulation experiments in a graph database

## Supplementary Data

files

**Files in this Data Supplement:**

- Supplementary Data - pdf file
- Supplementary Data - txt file
