# Supplementary material for: Combining computational models, semantic annotations and simulation experiments in a graph database
Source: Database (Oxford). 2015 Mar 8;2015:bau130. doi: 10.1093/database/bau130 (PMC4352687; doi:10.1093/database/bau130)
Supplement: Supplementary Data [file supp_bau130_WebAPI_and_Queries.pdf]

Web Interface

To try the queries described in the paper we provide access to the Noe4J cypher endpoint:

http://graphdb-review.sems.uni-rostock.de:17474/browser/

User: reviewer

Password: henkeletal

Every day at 2am (UTC+01:00) the database will be replaced by a backup, all changes are lost.

Queries

Here, we list all queries described in the paper. This makes it easier to copy and paste queries into the web interface mentioned above. Very helpful when writing queries is the Cypher reference card

Query 1

```
MATCH       (species:SBML_SPECIES)-[isMod:IS_MODIFIER]->()
WHERE       NOT((species)-[:IS_REACTANT]->() OR (species)-[:IS_PRODUCT]->())
WITH        species, count(isMod) AS numOfMod ORDER BY numOfMod DESC LIMIT 1
        MATCH species-[:BELONGS_TO]->model
        WHERE (model:SBML_MODEL)
RETURN model.NAME AS Model, species.NAME as Species, numOfMod
```

Query 1: Return the model with the most species acting only as a modifier.

Result 1: The model "Schaber2012 - Hog pathway in yeast" having the species Hog1PPActive which is acting as a modifier in ten reactions.

Query 2

```
MATCH       (m:SBML_MODEL)-[:REFERENCES_SIMULATION_MODEL]-ref-[:BELONGS_TO*2]
            ->(sed:DOCUMENT)
WHERE m.NAME='Novak1997 - Cell Cycle'
```

```
RETURN      m.NAME AS Model, m.ID as ModelID, ref.MODELSOURCE as ModelSource,
            sed.FILENAME as SEDMLFile
```

Query 2: Return all simulations that can be applied to the model "Novak1997 - Cell Cycle"

Result 2: The requested model can be run by two simulations, reproducing Figure 2a and 2b by Novak 97

Query 3

```
MATCH       (sed:DOCUMENT)<-[:BELONGS_TO*2]-(sim:SEDML_SIMULATION)-[:SIMULATES]

            ->(ref:SEDML_MODELREFERENCE)-[:REFERENCES_SIMULATION_MODEL]->m

WHERE       (sim.SIMKISAO='KISAO:0000019') AND

            filter(lable in labels(m) where lable ='CELLML_MODEL')

RETURN m.NAME, sed.FILENAME
```

Query 3: Return only CellML models that can be simulated using a Livermore Solver (KISAO:0000019).

Result 3: The CellML encoded "Tyson 1991" model and the corresponding SED-ML file.

Query 4

```
START       res=node:annotationIndex('RESOURCETEXT:(m-phase inducer phosphatase)')

MATCH       res<-[rel:is]-(a:ANNOTATION)-->(s:SBML_SPECIES) <-[:OBSERVES]-o-[:BELONGS_TO*]

            ->(doc:DOCUMENT)

WITH        doc,res,s,o

        MATCH ()<-[:IS_MODIFIER]-s-[:BELONGS_TO]->m

RETURN DISTINCT

            doc.FILENAME AS SEDML,

            collect(distinct m.NAME) AS Model,

            collect(distinct res.URI) AS Resource,

            collect(distinct s.NAME) AS Species,

            collect(distinct o.TARGET) AS Target
```

Query 4: Return simulation descriptions observing a particular species that plays the role of a modifier or reaction, respectively. The observed species should be annotated as "m-phase inducer phosphatase" using the qualifier is.

Result 4: The result is shown and explained in Figure 3.

Query 5

```
MATCH      (r:RESOURCE)-[qualifier:BELONGS_TO]->()
WITH       r, count(qualifier) AS AnnotationCount
     ORDER BY AnnotationCount DESC LIMIT 3
RETURN     r.URI as Annotation, AnnotationCount
```

Query 5: What are the top-most three annotations used

Result 5: Top three annotations used are SBO:0000009 (1127 times), SBO:0000252 (509 times), GO:0043241 (484 times)

Query 6

```
MATCH      ()-[rel]->(res:RESOURCE)-[:IS_ONTOLOGY_ENTRY]-c-[:isA*0..]->s
WHERE      s.id="SBO_0000009"
RETURN     count(rel)
```

Query 6: How many annotations point to the term SBO:0000009 or one of its children?

Result 6: 3373 annotations pointing to SBO:0000009 or one of its children, 1127 of them point directly to SBO:0000009.

Query 7

```
MATCH      (m:SBML_MODEL)<-[:BELONGS_TO*1..2]-(a:ANNOTATION)
           <-[:BELONGS_TO]-(r:RESOURCE)
WITH       m as Model, count(r) AS NumberOfAnnotation
RETURN     max(NumberOfAnnotation), min(NumberOfAnnotation),
           avg(NumberOfAnnotation), stdev(NumberOfAnnotation)
```

Query 7: What is the minimum, maximum and average number of annotations per model?

Result 7: A model has a maximum of 800, a minimum of three and an average of 71 annotations.

Query BM1

```
MATCH      (m:SBML_MODEL)-->(s:SBML_SPECIES)
WHERE      (m.ID="BIOMD0000000001")
RETURN     m AS Model, collect(s.ID) as SpeciesID, collect(s.NAME) as SpeciesName
```

Query BM1: From model BIOMD0000000001, list all species identifiers and names

Result BM1: 12 species IDs (ALL, I, DL, ILL, D, DLL, B, BL, A, AL, IL, BL) and names (ActiveACh2, Intermediate, …)

Query BM2

```
MATCH       (r:RESOURCE)-->()-[:BELONGS_TO]->(element)-->(m:SBML_MODEL)
WHERE       m.ID="BIOMD0000000001"
RETURN      element.ID AS Element, LABELS(element) AS ElmentType,
            collect(r.URI) AS ElementAnnotation
```

Query BM2: Get element annotations of the model BIOMD0000000001

Result BM2: 104 annotations for 65 distinct elements, for example species ALL is annotated with IPR002394, GO:0005892 and SBO:0000297

Query BM3

```
MATCH       (r:RESOURCE)<-[rel]-()-->e-[:BELONGS_TO]->(m:SBML_MODEL)
WHERE       r.URI=~".*GO.*0005892"
RETURN      m.ID AS ModelID, collect(e.ID) AS ElementIDs, type(rel) AS Qualifier, r.URI as URI
```

Query BM3: All model elements with annotations to acetylcholine-gated channel complex.

Result BM3: From each model (BIOMD0000000001 and BIOMD0000000002) the same 12 species IDs are returned (ALL, I, DL, ILL, D, DLL, B, BL, A, AL, IL, BL), all are qualified with isVersionOf. A graphical representation is shown in Figure~4.

Query P1

```
MATCH       (res:RESOURCE), (sbo:SBOOntology)
WHERE       (res.URI =~ ".*SBO.*") AND (RIGHT(res.URI, 7) = RIGHT(sbo.id, 7))
CREATE      res-[link:IS_ONTOLOGY_ENTRY]->sbo
RETURN      count(link);
```

Query P1: Select and match and link the SBO annotations extracted from models with corresponding concepts from the SB-Ontology.

Result P1: The number of created links.

Query M1

```
MATCH       (m:CELLML_MODEL)
```

```
RETURN          m
```

Query M1: Database look-up. Return all CellML models

Result M1: List of 841 models

Query M2

```
MATCH           (m:CELLML_MODEL)
WHERE           m.NAME = 'tyson_1991'
RETURN          m
```

Query M2: Database look-up and filtering. Return CellML models with the name "tyson_1991"

Result M2: A model node containing the attribute NAME:"tyson_1991"

Query M3

```
MATCH           (m:CELLML_MODEL)-->(c:CELLMLCOMPONENT)
WHERE           m.NAME = 'tyson_1991'
RETURN          c.NAME
```

Query M3: Database graph structure query. Select the aforementioned Tyson model and return all its components.

Result M3: The components YP, Y, M, pM, CP, C2, environment and reaction_constants.

Query M4

```
MATCH           (m:CELLML_MODEL)-->(c:CELLMLCOMPONENT)-->(v:CELLMLVARIABLE)
WHERE           m.NAME = 'tyson_1991'
RETURN          count(v)
```

Query M4: Database aggregation query. Count the number of variables contained by any component of the aforementioned Tyson model

Result M4: This model has 68 variables.

Query M5

```
MATCH           (m:CELLML_MODEL)-->(c:CELLMLCOMPONENT)-->(v:CELLMLVARIABLE)
WITH            c as component, count(v) as NumOfVar
RETURN          min(NumOfVar), max(NumOfVar), avg(NumOfVar), stdev(NumOfVar)
```

Query M5: Statistics query. Retrieve minimum, maximum average and standard derivation of for the number of variables attached to a component.

Result M5: A minimum of one and a maximum of 431 variables are attached to a component of a CellML model. On average each component has 9.64 variables attached with a standard derivation of almost 16.

Query M6

```
START     res=node:annotationIndex('RESOURCETEXT:(m-phase inducer phosphatase)')

RETURN    res
```

Query M6: Database index query. Retrieve all annotations containing the phrase "m-phase inducer phosphatase"

Result M6: A set of seven resources (InterPro IPR000751; Enzyme Commission number 3.1.3.48; and UniProt: P30311, P23748, P20483, P06652, P30304)

Nodes and Relationships

Neo4J connects two nodes by a relationship. Here we list all possible types of nodes and all relationships possible between two node types. This list is comparable to the database schema for relational databases.

Node	Relationship	Node
ANNOTATION	BELONGS_TO	MODEL
ANNOTATION	IS_CREATOR	PERSON
ANNOTATION	HAS_PUBLICATION	PUBLICATION
ANNOTATION	isDescribedBy	RESOURCE
ANNOTATION	is	RESOURCE
ANNOTATION	isVersionOf	RESOURCE
ANNOTATION	occursIn	RESOURCE
ANNOTATION	BELONGS_TO	SBML_COMPARTMENT
ANNOTATION	HAS_SBOTERM	RESOURCE
ANNOTATION	BELONGS_TO	SBML_SPECIES
ANNOTATION	BELONGS_TO	SBML_REACTION
ANNOTATION	BELONGS_TO	SBML_PARAMETER
ANNOTATION	BELONGS_TO	SBML_EVENT
ANNOTATION	isHomologTo	RESOURCE
ANNOTATION	hasVersion	RESOURCE
ANNOTATION	isDerivedFrom	RESOURCE
ANNOTATION	hasPart	RESOURCE
ANNOTATION	hasProperty	RESOURCE

ANNOTATION	encodes	RESOURCE
ANNOTATION	isPartOf	RESOURCE
ANNOTATION	BELONGS_TO	SBML_RULE
ANNOTATION	BELONGS_TO	SBML_FUNCTION
ANNOTATION	isEncodedBy	RESOURCE
CELLMLCOMPONENT	BELONGS_TO	MODEL
CELLMLCOMPONENT	HAS_VARIABLE	CELLMLVARIABLE
CELLMLCOMPONENT	IS_CONNECTED_TO	CELLMLCOMPONENT
CELLMLCOMPONENT	BELONGS_TO	CELLMLREACTION
CELLMLREACTION	HAS_REACTION	CELLMLCOMPONENT
CELLMLVARIABLE	BELONGS_TO	CELLMLCOMPONENT
CELLMLVARIABLE	IS_MAPPED_TO	CELLMLVARIABLE
CELLMLVARIABLE	HAS_DELTA_VAR	CELLMLVARIABLE
CELLMLVARIABLE	IS_DELTA_VAR	CELLMLVARIABLE
DOCUMENT	HAS_MODEL	MODEL
DOCUMENT	HAS_SEDML	SEDML
GOOntology	isA	GOOntology
KISAOOntology	isA	KISAOOntology
MODEL	BELONGS_TO	DOCUMENT
MODEL	HAS_ANNOTATION	ANNOTATION
MODEL	HAS_COMPONENT	CELLMLCOMPONENT
MODEL	HAS_REACTION	SBML_REACTION
MODEL	HAS_COMPARTMENT	SBML_COMPARTMENT
MODEL	HAS_SPECIES	SBML_SPECIES
MODEL	HAS_PARAMETER	SBML_PARAMETER
MODEL	HAS_EVENT	SBML_EVENT
MODEL	HAS_RULE	SBML_RULE
MODEL	HAS_FUNCTION	SBML_FUNCTION
PERSON	BELONGS_TO	PUBLICATION
PERSON	BELONGS_TO	ANNOTATION
PUBLICATION	BELONGS_TO	ANNOTATION
PUBLICATION	HAS_AUTHOR	PERSON
RESOURCE	BELONGS_TO	ANNOTATION
RESOURCE	IS_ONTOLOGY_ENTRY	GOOntology
RESOURCE	IS_ONTOLOGY_ENTRY	SBOOntology
SBML_COMPARTMENT	BELONGS_TO	MODEL
SBML_COMPARTMENT	HAS_ANNOTATION	ANNOTATION
SBML_COMPARTMENT	CONTAINS_SPECIES	SBML_SPECIES
SBML_EVENT	BELONGS_TO	MODEL
SBML_EVENT	HAS_ANNOTATION	ANNOTATION
SBML_FUNCTION	BELONGS_TO	MODEL
SBML_FUNCTION	HAS_ANNOTATION	ANNOTATION
SBML_PARAMETER	BELONGS_TO	MODEL

SBML_PARAMETER	HAS_ANNOTATION	ANNOTATION
SBML_REACTION	BELONGS_TO	MODEL
SBML_REACTION	HAS_ANNOTATION	ANNOTATION
SBML_REACTION	HAS_PRODUCT	SBML_SPECIES
SBML_REACTION	HAS_REACTANT	SBML_SPECIES
SBML_REACTION	HAS_MODIFIER	SBML_SPECIES
SBML_RULE	BELONGS_TO	MODEL
SBML_RULE	HAS_ANNOTATION	ANNOTATION
SBML_SPECIES	BELONGS_TO	MODEL
SBML_SPECIES	HAS_ANNOTATION	ANNOTATION
SBML_SPECIES	IS_LOCATED_IN	SBML_COMPARTMENT
SBML_SPECIES	IS_PRODUCT	SBML_REACTION
SBML_SPECIES	IS_REACTANT	SBML_REACTION
SBML_SPECIES	IS_MODIFIER	SBML_REACTION
SBOOntology	isA	SBOOntology
SEDML	BELONGS_TO	DOCUMENT
SEDML	HAS_MODELREFERENCE	SEDML_MODELREFERENCE
SEDML	HAS_SIMULATION	SEDML_SIMULATION
SEDML	HAS_TASK	SEDML_TASK
SEDML	HAS_DATAGENERATOR	SEDML_DATAGENERATOR
SEDML	HAS_OUTPUT	SEDML_OUTPUT
SEDML_CURVE	BELONGS_TO	SEDML_OUTPUT
SEDML_DATAGENERATOR	BELONGS_TO	SEDML
SEDML_DATAGENERATOR	HAS_VARIABLE	SEDML_VARIABLE
SEDML_MODELREFERENCE	BELONGS_TO	SEDML
SEDML_MODELREFERENCE	IS_REFERENCED_IN_TASK	SEDML_TASK
SEDML_MODELREFERENCE	IS_SIMULATED	SEDML_SIMULATION
SEDML_MODELREFERENCE	USED_IN_DATAGENERATOR	SEDML_VARIABLE
SEDML_MODELREFERENCE	REFERENCES_SIMULATION_MODEL	MODEL
SEDML_OUTPUT	BELONGS_TO	SEDML
SEDML_OUTPUT	HAS_CURVE	SEDML_CURVE
SEDML_SIMULATION	BELONGS_TO	SEDML
SEDML_SIMULATION	IS_ONTOLOGY_ENTRY	KISAOOntology
SEDML_SIMULATION	IS_REFERENCED_IN_TASK	SEDML_TASK
SEDML_SIMULATION	SIMULATES	SEDML_MODELREFERENCE
SEDML_TASK	BELONGS_TO	SEDML
SEDML_TASK	REFERENCES_MODEL	SEDML_MODELREFERENCE
SEDML_TASK	REFERENCES_SIMULATION	SEDML_SIMULATION
SEDML_VARIABLE	BELONGS_TO	SEDML_DATAGENERATOR
SEDML_VARIABLE	CALCULATES_MODEL	SEDML_MODELREFERENCE
SEDML_VARIABLE	OBSERVES	SBML_SPECIES
SEDML_VARIABLE	OBSERVES	CELLMLVARIABLE

Rest API

The RestApi is not described in this paper. For more information, please refer to:

https://sems.uni-rostock.de/projects/morre/

Please keep in mind that the database described on the webpage above is only available for REST access. It is not the same database, password protected, database specifically setup for the review process.
